# The role of *lin-12 notch* in *C. elegans* anchor cell proliferation

**DOI:** 10.1242/bio.061816

**Published:** 2024-12-30

**Authors:** Alex Hajnal, Ting Deng, Evelyn Lattmann

**Affiliations:** ^1^ Department of Molecular Life Sciences, University of Zurich, Winterthurerstrasse 190, Zürich CH-8057, Switzerland; ^2^ JingAn Kerry Center, 1228 Middle Yan An Road, Shanghai, China, 200040; ^3^ Department of Dermatology, University of Zurich, University Hospital Zurich, Schlieren CH-8952, Switzerland

**Keywords:** *lin-12 notch*, *C. elegans*

## Abstract

The gonadal anchor cell (AC) is an essential organizer for the development of the egg-laying organ in the *C. elegans* hermaphrodite. Recent work has investigated the mechanisms that control the quiescent state the AC adopts while fulfilling its functions. In this context, the transcription factors EGL-43 and NHR-67 are required to maintain the G1 cell cycle arrest of the AC and prevent proliferation. While NHR-67 acts primarily by up-regulating the CDK inhibitor CKI-1, the role of EGL-43 in this process has been subject to debate. Deng et al. (2020) reported that inhibition of the *notch* receptor *lin-12* by RNAi partially suppressed the AC proliferation phenotype caused by *egl-43* RNAi. By contrast, Martinez et al. (2022) found that down-regulation of LIN-12 NOTCH via the auxin-inducible degradation system did not reduce AC proliferation. To resolve this issue, we performed *egl-43* RNAi in the background of a *lin-12* null allele and observed a similar suppression of AC proliferation as reported previously by Deng et al. (2020). Hence, AC proliferation caused by the downregulation of *egl-43* partially depends on LIN-12 NOTCH signaling.

## Background

The gonadal anchor cell (AC) in *C. elegans* hermaphrodites is specified at the beginning of the second larval stage (L2) from one of two equivalent precursor cells (Z1.ppp and Z4.aaa) (Greenwald, 2005). Reciprocal *lin-12 notch* signaling between the two AC precursors determines the AC fate. The precursor cell exhibiting higher LIN-12 NOTCH activity adopts the ventral uterine (VU) cell fate, while the cell with lower LIN-12 NOTCH activity adopts the default AC fate. Hence, if LIN-12 NOTCH signaling is inactive at this stage, two ACs are specified at the expense of a VU cell (Greenwald et al., 1983). After its specification, the AC permanently arrests in the G1 phase of the cell cycle. Maintaining the AC arrested in G1 is necessary for its later AC functions during vulval development, especially for breaching the basement membranes (BMs) during AC invasion at the late L3 stage (Matus et al., 2015). The two transcription factors NHR-67, a nuclear receptor of the *tailless* family, and EGL-43, a homolog of the mammalian EVI1 proto-oncogene, are required to keep the AC arrested in the G1 phase (Medwig-Kinney et al., 2020; Deng et al., 2020). NHR-67 acts primarily by up-regulating the expression of the CDK inhibitor CKI-1. A search for EGL-43 targets identified the *notch* receptor *lin-12* as a gene repressed by EGL-43. Loss of *eg-43* resulted in an up-regulation of *lin-12* expression in the AC and expression of a constitutively active LIN-12 intracellular fragment (NICDΔCT) induced AC proliferation (Deng et al., 2020; Martinez et al., 2022). However, there are conflicting reports on the consequences of inhibiting LIN-12 NOTCH signaling in the AC. While Deng et al. (2020) found that simultaneous inhibition of *egl-43* and *lin-12* by RNAi prevented AC proliferation, Martinez et al. (2022) reported that down-regulation of LIN-12 through the auxin-inducible degradation system (AID) did not suppress AC proliferation.

## Results and discussion

**Fig. 1.**
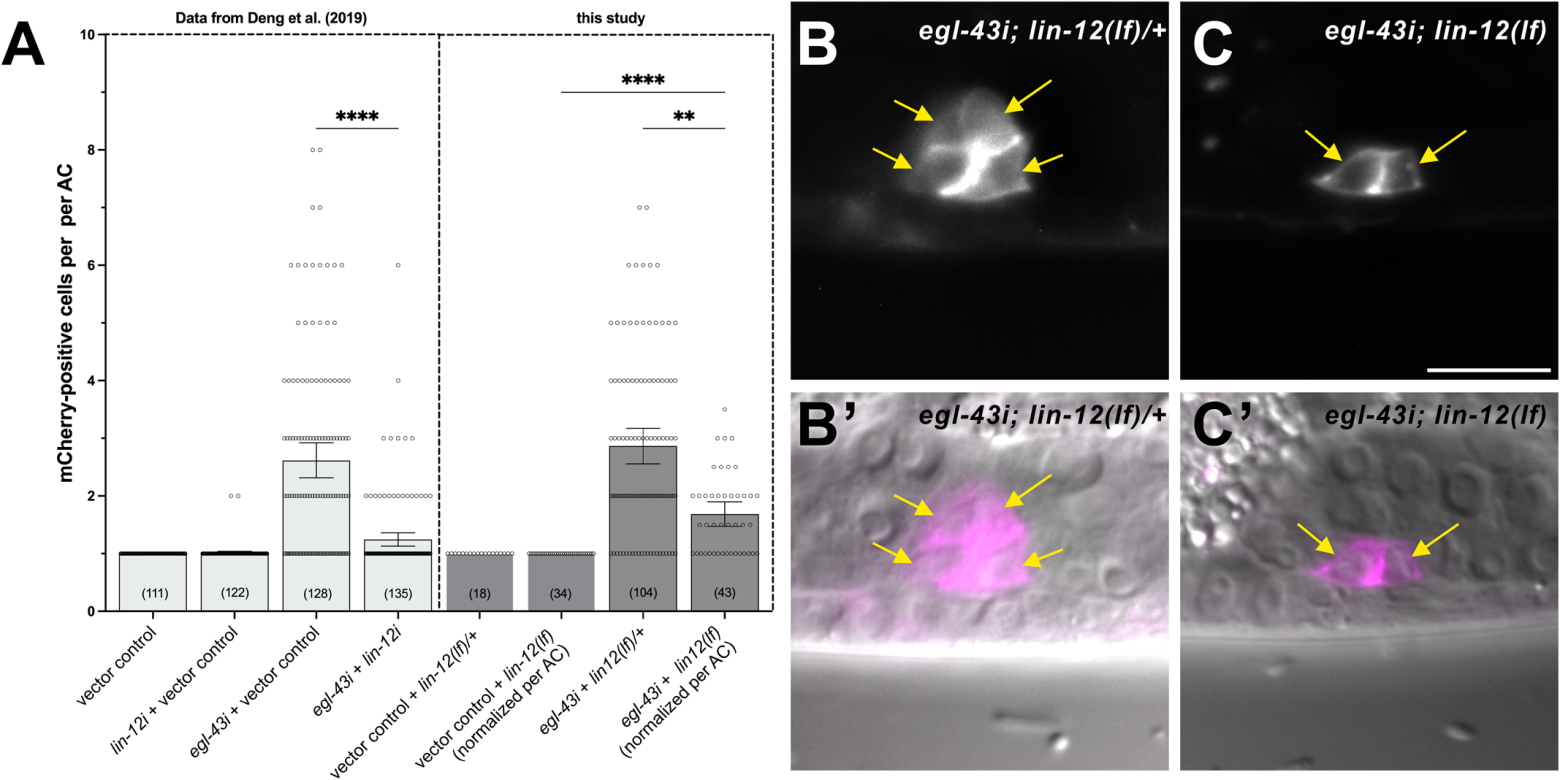
**AC proliferation in *lin-12* RNAi animals versus *lin-12(lf)* mutants with *egl-43* RNAi.** (A) Quantification of AC proliferation after *egl-43* & *lin-12* double RNAi (left half, data from Deng et al., 2020) and *egl-43* RNAi in heterozygous versus homozygous *lin-12(lf)* mutants (right half). An empty RNAi vector was used for the negative controls. mCherry-positive cell numbers in homozygous *lin-12(lf)* mutants were normalized to the initial number of two ACs formed during early L2. Error bars indicate 95% confidence intervals and numbers in brackets the numbers of animals analyzed per condition. ****P<0.0001 and **P<0.01. The data are from three biological replicates, except for two replicates for the vector control in the *lin-12(lf)* background. See Table S1 for the statistical tests used. (B) Example of a heterozygous *egl-43i, lin-12(lf)/+* animal containing four cells expressing the *Pcdh-3>mcherry::PLCdeltaPH* AC marker (*qyIs24*) and (C) a homozygous *egl-43i, lin-12(lf)* animal, in which the two AC formed during early L2 that did not proliferate. (B′,C′) The bottom panels show DIC images overlaid with the mCherry signal. The yellow arrows point to the AC nuclei. The scale bar in C is 10 µm.

To resolve these conflicting observations, we used the *lin-12(lf)* (null) allele *(n137n720)* (Greenwald et al., 1983) in combination with *egl-43* or vector control RNAi and scored AC proliferation at the late L3 (Pn.pxx) stage. Since *lin-12(lf)* results in the specification of two ACs during the early L2 stage, the numbers of proliferating ACs in *lin-12(lf)* mutants were normalized to the two ACs initially formed per animal. Our results indicated that a complete loss of *lin-12* function reduces AC proliferation during the L3 stage to a similar degree as *lin-12* RNAi (Fig. 1). However, neither *lin-12(lf)* nor *lin-12* RNAi completely blocked AC proliferation, indicating that EGL-43 prevents AC proliferation not exclusively by repressing *lin-12* expression.

Thus, a complete loss or strong down-regulation of *lin-12* is required to reduce AC proliferation caused by inhibiting *egl-43*. AID-mediated degradation of LIN-12 may leave sufficient residual LIN-12 activity for the AC to proliferate. The observation that LIN-12 AID causes an early AC duplication phenotype (Martinez et al., 2022) could indicate that relatively strong LIN-12 NOTCH signaling in late L1 and early L2 larvae is necessary to prevent specification of the default AC cell fate. Hence, a partial reduction in LIN12 activity may be sufficient to permit both precursor cells to differentiate into ACs but not to prevent AC proliferation. Finally, it should be noted that not in every case reported AC proliferation depended on *lin-12 notch* activity. For example, *lin-12* RNAi did not suppress the AC proliferation phenotype caused by *nhr-67* RNAi (Deng et al., 2020), and overexpression of the *hox* gene *lin-39* induced AC proliferation independently of *lin-12* (Heinze et al., 2023), pointing to a variety of mechanisms that maintain the quiescent state of the AC.

## Materials and methods

Strain used: AH6848, genotype: +/*hT2[bli-4(e937) let(q782) qIs48] (I;III); rrf-3(pk1426) II; unc-32(e189) lin-12(n137n720) III/ hT2[bli-4(e937) let(q782) qIs48] (I;III); qyIs10 IV; qyIs24 X*.

### RNA interference

The RNAi clone was obtained and sequence-verified from a *C*. *elegans* genome-wide RNAi library (Source BioScience). Bacteria were grown overnight in 2 ml of LB medium containing 200 µg/ml ampicillin and 25 µg/ml tetracycline at 37°C, diluted 1:100 in LB medium containing the antibiotics and 1 mM IPTG, and grown for another 4 to 6 h at 37°C before seeding them on NGM plates containing 1 mM IPTG. Embryos from strain AH6848 were isolated by hypochlorite treatment of gravid adults and allowed to hatch overnight in M9 buffer to obtain synchronized L1 larvae that were plated on NGM plates seeded with *E. coli* producing *egl-43* dsRNA or containing the empty RNAi vector. Animals were grown for 38 to 42 h at 20°C until late L3 when they were analyzed. Heterozygous *lin-12(lf)/+* siblings grown on the same RNAi plates as the *lin-12(lf)* homozygotes were used as controls.

### Microscopy

Nomarski and fluorescent images of late L3 larvae at the Pn.pxx stage (before vulval invagination) were acquired with a LEICA DM6000B microscope equipped with a Hammamatsu ORCA FLASH 4.0LT sCMOS camera, a piezo objective drive (Piezosystems Jena, Germany) and a 63x (N.A. 1.32) oil-immersion lens. z-stacks were acquired with a spacing of 0.5 μm, and images were deconvolved using the YacuDecu implementation of CUDA-based Richardson Lucy deconvolution in MATLAB (www.github.com/bobpepin/YacuDecu). ACs were counted across the z-stacks using the *qyIs24[Pcdh-3>mcherry::PLCdeltaPH]* membrane marker (Ziel et al., 2008) overlaid on the Nomarski channel to visualize the nuclei.

## Supplementary Material

10.1242/biolopen.061816_sup1 Supplementary information

Table S1.
