# Supplementary material for: The role of lin-12 notch in C. elegans anchor cell proliferation
Source: Biol Open. 2024 Dec 30;13(12):bio061816. doi: 10.1242/bio.061816 (PMC11708767; doi:10.1242/bio.061816)
Supplement: Supplementary information [file biolopen-13-061816-s1.pdf]

Table S1.

Available for download at
https://journals.biologists.com/bio/article-lookup/doi/10.1242/bio.061816#supplementary-data
